# Architecture of a Serine Recombinase-DNA Regulatory Complex

**DOI:** 10.1016/j.molcel.2008.02.023

**Published:** 2008-04-25

**Authors:** Kent W. Mouw, Sally-J. Rowland, Mark M. Gajjar, Martin R. Boocock, W. Marshall Stark, Phoebe A. Rice

**Affiliations:** 1 Department of Biochemistry and Molecular Biology, The University of Chicago, Chicago, IL 60637, USA; 2 Division of Molecular Genetics, FBLS, University of Glasgow, Glasgow G12 8QQ, Scotland, UK

**Keywords:** DNA

## Abstract

An essential feature of many site-specific recombination systems is their ability to regulate the direction and topology of recombination. Resolvases from the serine recombinase family assemble an interwound synaptic complex that harnesses negative supercoiling to drive the forward reaction and promote recombination between properly oriented sites. To better understand the interplay of catalytic and regulatory functions within these synaptic complexes, we have solved the structure of the regulatory site synapse in the Sin resolvase system. It reveals an unexpected synaptic interface between helix-turn-helix DNA-binding domains that is also highlighted in a screen for synapsis mutants. The tetramer defined by this interface provides the foundation for a robust model of the synaptic complex, assembled entirely from available crystal structures, that gives insight into how the catalytic activity of Sin and other serine recombinases may be regulated.

## Introduction

**Figure 1:**
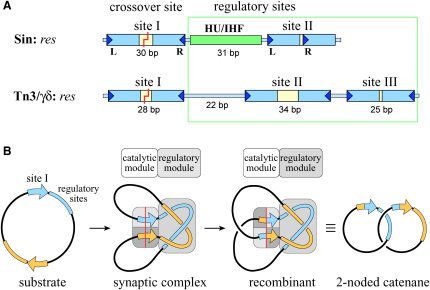
*res* Site Architecture and Recombination Topology (A) *res* site architecture. In the Sin system, each *res* site binds two dimers of Sin and an architectural protein such as HU or IHF (at the synthetic *resF* site). The *res* sites from the Tn3/γδ systems are longer than the Sin *res* site (114 versus 86 bp) and bind three separate recombinase dimers and no architectural proteins. In both systems, DNA cleavage occurs within site I to generate 2 nt 3′ overhangs, while the regulatory sites (site II and the HU/IHF binding site in the Sin system and sites II and III in the Tn3/γδ systems) serve to stimulate recombination and direct the outcome of the reaction. (B) Recombination topology. Sin resolves plasmid dimers to monomers by recombining two *res* sites that are present in direct repeat on a supercoiled plasmid. The synaptic complexes assembled by Sin and by Tn3/γδ resolvase trap three (−) interdomainal supercoils, and the predominant reaction product is a two-noded catenane.

Site-specific recombination reactions take place within intricate synaptic complexes in which four or more subunits of recombinase reorganize the two DNA target sites, often with the help of accessory proteins (Biswas et al., 2005; Chen et al., 2000; Grindley et al., 2006; Guo et al., 1997; Li et al., 2005; MacDonald et al., 2006). Because these reactions lack high-energy cofactors and are chemically isoenergetic, catalysis is regulated in diverse systems by a separate regulatory module within the synaptic complex, which uses topological cues to direct recombination toward a biologically relevant outcome (Figure 1) (Grindley et al., 2006; Stark and Boocock, 1995). However, efforts to understand the architecture of the synaptic complexes and the molecular basis for regulation have been frustrated by a lack of structural information regarding the regulatory modules (Li et al., 2005; Murley and Grindley, 1998).

Sin is a resolvase of the serine recombinase family that is encoded by various *S. aureus* multiresistance plasmids (Paulsen et al., 1994; Rowland and Dyke, 1989). It is thought to stabilize plasmids by resolving dimers into monomers. The Sin synaptic complex brings together two directly oriented 86 bp *res* sites on the same supercoiled plasmid, trapping three highly condensed supercoils (Rowland et al., 2002), as in the related Tn3/γδ resolvase systems (Wasserman et al., 1985). Each *res* site binds two dimers of Sin, at sites I and II, and a nonspecific DNA-bending protein, e.g., HU (Figure 1) (Rowland et al., 2002). IHF, a site-specific DNA-bending protein that is 30% identical to HU, can substitute for HU with no loss of function if a cognate site is placed precisely at the center of the site I-site II spacer (Rowland et al., 2006). DNA cleavage and strand exchange take place at the center of site I, where two subunits of Sin form phosphoserine linkages with the DNA to create a double-strand break. Site II differs from site I (and from the regulatory sites in the Tn3/γδ system) in its head-to-tail configuration of Sin-binding motifs (Figure 1) (Rowland et al., 2002). Site II and the DNA-bending protein are both needed to activate the catalytic machinery at site I (Rowland et al., 2005).

Synapsis of the regulatory Sin dimers at site II is thought to initiate assembly of the synaptic complex, leading to formation of an active catalytic tetramer at site I (Rowland et al., 2002). Crystal structures of the related (32% identical) γδ resolvase show that the transition from the inactive precleavage site I dimer to the postcleavage tetramer is accompanied by dramatic conformational rearrangements, resulting in formation of a high-symmetry (222) complex with the C-terminal DNA-binding domains (CTDs) and cleaved DNA on the outside (Kamtekar et al., 2006; Li et al., 2005; Yang and Steitz, 1995). A flat, hydrophobic surface within the synapsed N-terminal catalytic domains is thought to facilitate subunit rotation and DNA exchange (Dhar et al., 2004; Li et al., 2005).

The overall architecture of the synaptic complex and the regulatory interactions are poorly understood: published ideas (Murley and Grindley, 1998; Rice and Steitz, 1994a; Rowland et al., 2002; Sarkis et al., 2001) are difficult to reconcile with the structural data for site I (Kamtekar et al., 2006; Li et al., 2005). To address these issues, we have solved the crystal structure of a wild-type (WT) Sin dimer bound to a site II duplex. This crystal structure of a serine recombinase bound to a regulatory site has transformed our understanding of the regulatory module. Site II synapsis is mediated not by the catalytic domains (as in site I synapsis) but by a previously undescribed interface between DNA-binding domains. Docking of the Sin-site II synaptic tetramer with existing γδ resolvase site I and IHF-DNA crystal structures leads to a remarkably plausible model for the complete Sin synaptic complex that accounts for the reaction topology and suggests how the regulatory sites might control the catalytic activity at site I.

## Results

### Structure of the Sin-Site II Dimer

**Figure 2:**
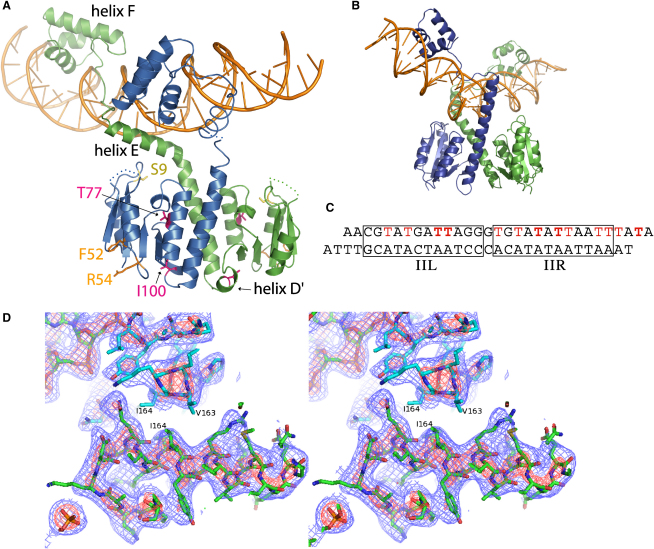
Crystal Structure of the Site II-Bound Sin Dimer (A) Individual Sin monomers are shown in blue and green; the site II duplex is in orange. Ser-9, the active site nucleophile at site I, is shown in yellow. The positions of activating (T77I, I100T) and regulatory (F52, R54) mutations highlighted in the genetic screens (see Figure 5) are shown in magenta and orange, respectively. Residues 35–41 from both monomers, as well as residues 130 and 131 from the blue monomer, are not present in the final model but are represented here with dotted lines. (B) Structure of the γδ resolvase site I dimer complex (Yang and Steitz, 1995). (C) The sequence of the site II duplex in the crystal. The left and right half-sites of the site II direct repeat are boxed. T's shown in red were substituted with 5-Br-dU, and bold T's were substituted with 5-I-dU. (D) Stereo-view experimental electron density map showing interface between the CTDs of adjacent Sin dimers in the crystal. Residues V163 and I164 are at the center of the interface (see text). The maps contain no model phase information and are contoured at 2.5 σ (red) and 1.0 σ (blue).

WT Sin was cocrystallized with a 29 bp DNA duplex containing the site II direct repeat (Figure 2) (Rowland et al., 2002). Each duplex is bound by a dimer of Sin and forms a pseudocontinuous helix with adjacent duplexes in the crystal via base pairing of overhanging AT dinucleotides. Experimental phases were determined by using a combination of MAD and SIRAS (Figure 2, see Experimental Procedures). The 3.21 Å resolution model has been refined to an R_working_ of 26.7% and an R_free_ of 29.1% with good stereochemistry (Table 1 and Figure S4, available online).

Each Sin monomer is composed of a mixed αβ N-terminal catalytic domain and a helix-turn-helix (HTH) CTD that are linked through an extended helix (helix E), which forms the majority of the dimerization interface (Figure 2). Although the overall fold of Sin is similar to that of γδ resolvase, particularly the inactive WT dimer bound to an isolated site I (Rice and Steitz, 1994b; Sanderson et al., 1990; Yang and Steitz, 1995), several notable differences exist (Figure 2). β strand 5 of γδ resolvase is replaced in Sin by a short α helix, which we denote as helix D′; the orientation of helix D′ relative to the rest of the catalytic domain varies between individual monomers. This region may be involved in controlling the position of helix E, which shifts during formation of the proposed rotation interface in the γδ resolvase site I postcleavage tetramer (Kamtekar et al., 2006; Li et al., 2005). Helix D′ and the N-terminal portion of helix E are unusually rich in methionines, whose flexibility may accommodate this conformational change. Interestingly, helix D′ is also the location of the activating mutation I100T (Figure S2) (Rowland et al., 2005). Compared to γδ resolvase, Sin also has an 11 residue insertion at the beginning of its CTD, part of which forms an additional turn at the N terminus of helix F (Figure S2).

The site II-bound Sin dimer is markedly asymmetric, reflecting the requirement for the CTDs to dock with DNA repeat motifs in a head-to-tail configuration. The N-terminal catalytic domains are related by an imperfect two-fold rotation; however, the E helices bend sharply in different directions as they emerge from the catalytic domains. The chains then track along the same face of the DNA and deliver the CTDs into the major groove nearer to the catalytic domain of the other monomer (Figure 2). This is in contrast to the γδ resolvase site I dimer complex, where the E helices track through the minor groove on opposite faces of the DNA, the CTDs are docked nearer to their respective catalytic domains, and the DNA is oriented very differently relative to the dimer (Yang and Steitz, 1995). As seen in γδ resolvase site I complexes, insertion of the C-terminal portion of helix E into the minor groove bends the DNA away from the catalytic domains. However, in the Sin-site II complex, the bends in helix E are more drastic and asymmetric than those seen in γδ-site I complexes and only one E helix is inserted into the minor groove.

Sin bound to site II is catalytically inactive. The catalytic serines are >12 Å from the DNA and are partially blocked from it by the E helix of the other monomer. This arrangement of the E helices is necessitated by the head-to-tail orientation of site II half-sites and is incompatible with the mode of strand cleavage and exchange proposed for the site I tetramer (Dhar et al., 2004; Li et al., 2005).

### Tetrameric Interactions

WT Sin exists as a dimer in solution (K.W.M. and P.A.R, unpublished data) but can synapse two isolated segments of DNA bearing site II, presumably through formation of a stable tetramer (Rowland et al., 2002). There are three distinct interfaces between site II-bound Sin dimers within the crystal, two of which bury sufficient surface area (967–1865 Å^2^) to be plausible candidates for the synapsis interface and do not resemble any interfaces previously observed in serine recombinase structures.

**Figure 3:**
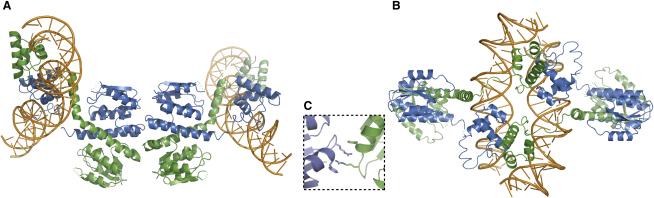
Tetrameric Interfaces Observed in the Crystal Structure (A) An interface involving the N-terminal catalytic domains places the bound site II duplexes along the outside of the complex. The duplexes define a right-handed (+) node crossing; this structure is thus a poor candidate for the site II synaptic complex. (B) The interface between DNA-binding domains defines a tetramer in which the bound duplexes are near the center of the complex and cross to form a left-handed (−) node. The orientation and close proximity of the duplexes make this a good candidate for the site II synaptic interface. (C) A close-up view of the interdigitating interaction involving the side chains of residues F52 and R54 from two adjacent dimer complexes in the crystal structure (see also Figure S1).

One tetramer, formed by pseudosymmetric interactions between catalytic domains (Figure 3A), is similar to the activated γδ resolvase-site I tetramer in that the DNA lies on the periphery of the complex; it more closely resembles the hypothesized synaptic tetramer of Sarkis et al. (2001). However, topological data suggest that synapsis at site II will create a left-handed (−) node, while in this structure, the duplexes cross to form a right-handed (+) node. The large distance between the site II duplexes (∼80 Å) also makes it difficult to construct a satisfactory model for the entire synaptic complex (Figure S3). Therefore, the tetramer defined by this interface is unlikely to mediate site II synapsis; however, it may provide a useful model for the site I tetramer prior to activation.

**Figure 4:**
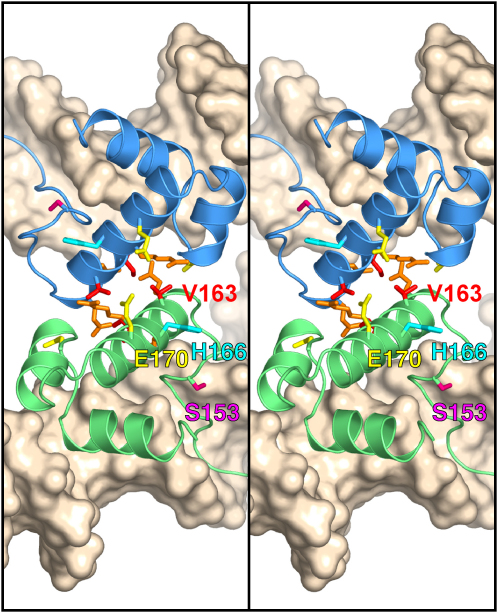
Stereo View of the Interface between Sin DNA-Binding Domains in the Site II Synaptic Tetramer Residues from helix F comprise much of the interface. Side chains are shown for all residues that, when mutated, confer a defect in synapsis and recombination (see Figure 5). Red, V163 and I164; orange, Q160, K161, and R167; yellow, E170 and N186; and magenta, S153. Also shown is H166 (cyan), the position of the suppressor mutation H166R. The DNA-binding domains of Sin subunits bound at site IIL (green) and site IIR (blue) are shown (N-terminal domains not shown).

The second potential tetramer interface buries substantially more surface area (1865 Å^2^) and is composed entirely of contacts between the DNA-binding domains (CTDs), primarily the N-terminal portions of each helix F (Figures 3B and 4). The distinctive geometry of site II aligns the binding domains such that both pairs of CTDs interact similarly—thus, site II-bound dimers can form cooperative CTD-mediated interactions. The Sin tetramer defined by this interface places the binding domains and associated DNA duplexes near the center of the complex with the catalytic domains on the outside. The duplexes cross each other at a ∼60° angle to form a negative node and are <10 Å apart. Because each DNA duplex is bent away from the body of the dimer to which it is bound, the tetramer would trap a highly condensed (−) node in supercoiled DNA. The CTD interface is thus a plausible candidate for a synaptic interaction, and this is strongly supported by the mutational data and modeling described below.

**Figure 5:**
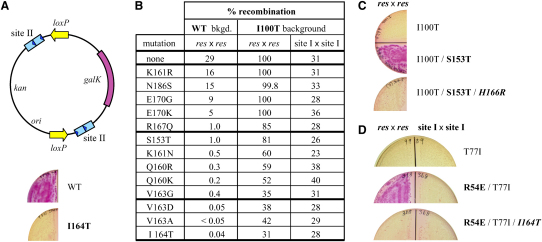
Effects of CTD Synapsis Mutations and R54E on the Regulation of Sin Recombination (A) Substrate plasmid used to select site II synapsis mutants in vivo, and site II synapsis assays for WT Sin and I164T. Site II synapsis by WT Sin blocks Cre-*loxP* recombination, preventing loss of the *galK* gene (red colonies on indicator plates); synapsis mutants (e.g., I164T) fail to block Cre (white colonies). (B) Quantitative recombination assays. The listed mutations were selected as causing a defect in site II synapsis in a WT background. The effect of each mutation on recombination is shown in a WT background (after ∼57 generations of growth) and in an I100T background (after ∼28 generations). Note that values of 100% recombination, seen in the I100T background, represent saturation of the assay. (C) H166R was selected as a second-site mutation that suppresses the inhibitory effect of S153T on *res* × *res* recombination in an I100T background. White colonies indicate that most or all of the test substrate has been resolved after ∼20 generations of growth; red colonies indicate inhibition of recombination. (D) The mutation R54E selectively inhibits *res* × *res* recombination, and the effect can be suppressed by a CTD mutation (e.g., I164T). Recombination was assayed in a T77I background; T77I is an activated mutant of Sin that can recombine site I × site I substrates (S.J.R., unpublished data). Inhibition by R54E requires site II in both recombining sites (data not shown), suggesting that site II synapsis is required.

The third interdimer interface is less extensive and primarily involves the side chains from two residues in the catalytic domain, F52 and R54, the hydrophobic portions of which form an interdigitated stack (Figure 3C and Figure S1). These residues are well conserved among many resolvases (Figure S2), and data implicating R54 in regulation of Sin activity are described below (Figure 5D). A strikingly similar arrangement of γδ resolvase dimers occurs across the so-called 2-3′ crystallographic interface (Figure S1); two of the residues involved (K54 and E56) align with F52 and R54 in Sin (Figure S2) (Hughes et al., 1990; Rice and Steitz, 1994b). Mutations in the 2-3′ interface disrupt communication between the catalytic and regulatory sites (Murley and Grindley, 1998). We suggest that the interface observed in the Sin crystal structure represents an analogous interaction that links catalytic and regulatory subunits in the synaptic complex.

### Genetic Analysis of Site II Synapsis

Evidence that Sin CTD interactions are necessary and sufficient for synapsis at site II was obtained by random mutagenesis of the Sin reading frame. Mutants that are defective in site II synapsis, but remain proficient in site II binding, were selected in two steps by using reporter plasmids. Synapsis was detected in vivo by its ability to interfere with the excision of a reporter gene by Cre-*loxP* recombination, when site II is located adjacent to each *loxP* site (Figure 5A; see Experimental Procedures). This assay is similar in principle to an in vitro assay described for Tn3 resolvase (Kilbride et al., 1999). The synapsis mutations mapped exclusively to the CTD: 13 substitutions in eight different residues were isolated (Figure 5B, column 1). Six of these residues, located on one face of helix F, plus Asn-186 at the C terminus of helix G, define a surface that closely matches the crystallographic CTD interface (Figure 4). Ser-153 is not on the interface but might contribute to synapsis by positioning helix F.

The CTD interface is important for recombination. All of the mutants selected as synapsis defective were also defective in *res* × *res* recombination (Figure 5B, column 2). Substitutions at residues 163 and 164, which form the hydrophobic core of the interface (Figure 4), had the strongest effects (e.g., I164T reduced recombination by ∼750-fold).

To demonstrate that CTD-mediated synapsis is specific to site II and is required to stimulate recombination at site I, we made use of the activated Sin mutant I100T. Unlike WT Sin, this enzyme recombines isolated site Is at a low level, although it is strongly stimulated when site II and HU are present and an interwound synaptic complex can assemble (i.e., in *res* × *res* recombination; Figure 1) (Rowland et al., 2002). None of the CTD mutations significantly inhibited site I × site I recombination, assayed in an I100T background (Figure 5B, column 4). However, mutations at residues 163 and 164 abolished the site II-dependent stimulation of recombination; other CTD mutations reduced the level of stimulation (e.g., S153T) or had no discernible effect (e.g., K161R) (Figure 5B, column 3). Because these inhibitory effects correlate well with those seen in the WT background (Figure 5B, column 2), we deduce that CTD-mediated synapsis at site II is essential for activity of the WT Sin synaptic complex.

Further evidence that the CTD interface is critical for regulated recombination was obtained by selecting for second-site mutations that rescue the recombination defect associated with S153T (see Experimental Procedures). The suppressor mutation identified, H166R, restored *res* × *res* recombination by I100T/S153T Sin (Figure 5C) and partially restored site II synapsis (data not shown); it also rescued the recombination defect of S153T Sin (data not shown). His-166 is adjacent to the CTD interface (Figure 4), and extra contacts between Arg-166 and Glu-170 across the interface could potentially reinforce the synapse. Consistent with this idea, we have evidence that H166R slightly activates *res* × *res* recombination by WT Sin (data not shown).

To investigate whether residue R54 is involved in communication between sites I and II, we constructed R54E Sin (R54 aligns with the γδ resolvase 2-3′ regulatory residue E56 [Figure S2]; Murley and Grindley, 1998). In a hyperactive Sin background (T77I), R54E selectively inhibited *res* × *res* recombination (i.e., when site II was present in both recombining sites; Figure 5D). (In a WT background, R54E reduced recombination by ∼1000-fold [data not shown].) We predicted that the inhibitory effect of R54E would require synapsis at site II and would thus be abolished by mutating the CTD interface; this was observed (Figure 5D). These data are consistent with the idea that a CTD-mediated synaptic tetramer at site II normally stimulates events at site I in a process requiring R54 but can inhibit these same events if R54 is mutated.

### A Model for the Sin Synaptic Complex

**Figure 6:**
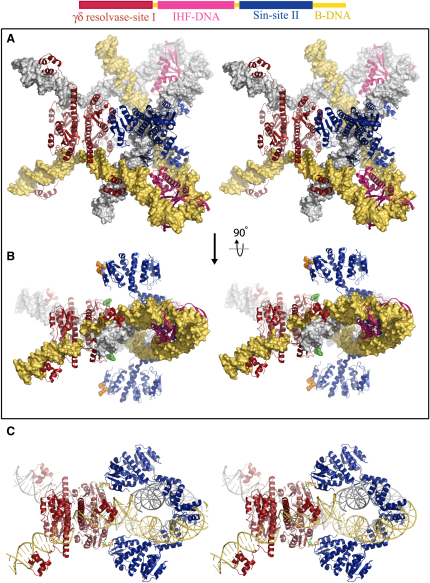
A Model for the Sin Synaptic Complex in Stereo View (A) The model was constructed by rigid body docking of the Sin-site II synaptic tetramer (Figure 3B) with existing crystal structures of the IHF-DNA (Rice et al., 1996) and γδ resolvase-site I tetramer (Li et al., 2005) complexes. A 12 bp segment of canonical B form DNA has been added at the end of each site II in order to better visualize the path of the DNA as it exits the synaptic complex. No steric clashes between protein subunits are observed, and the DNA forms the three (−) nodes predicted by topological experiments. The position and orientation of the IHF site are as in *resF^D^* (Rowland et al., 2006). (B) A second view of the synaptic complex model rotated 90° about a horizontal axis. Residues implicated in intertetramer communication are highlighted: Sin F52 and R54 at site II are shown in orange, and the equivalent positions in γδ resolvase at site I (residues 54 and 56) are shown in green. (C) A direct interaction between Sin bound at sites I and II can be modeled by using the observed crystallographic interface involving residues F52 and R54 (Figure 3C). The NTDs of the site I-bound γδ tetramer have been replaced with Sin NTDs, and IHF-DNA complexes have been removed from the model for clarity. Sin residues F52 and R54 from sites I and II are shown in green and orange, respectively. Unlike the model in (A) and (B), adjustments to the protein are required to construct this model and result in small gaps in the protein (at the hinge region of the site II-bound Sin dimers, shown in pink) and the DNA (the spacer bound by IHF, not shown, does not completely bridge the site I–site II gap).

The Sin-site II tetramer structure (Figure 3B) provides the foundation for a complete model of a resolvase synaptic complex that is based entirely on existing crystal structures (Figure 6), because the individual structures of the IHF-DNA complex (Rice et al., 1996) and a postcleavage γδ resolvase-site I tetramer (Kamtekar et al., 2006; Li et al., 2005) have already been solved. IHF rather than HU was used in model building because it provides additional important constraints. First, IHF's sequence specificity allowed its precise register relative to the Sin dimers to be determined (Rowland et al., 2006). Second, the 35 bp DNA in the IHF structure can be overlapped with the DNAs in the γδ resolvase-site I and Sin-site II structures, whereas the DNAs in available HU complex structures are too short to bridge the site I-site II gap.

The three protein-DNA complexes were treated as rigid bodies (the γδ resolvase-site I tetramer was first slightly adjusted by symmetrizing the DNA-bound dimers) and docked together, overlapping the extra DNA base pairs from adjacent complexes to attain the exact length and register of the 86 bp *res* site. The IHF-DNA complex was positioned within *res* in the register that allows efficient recombination (Rowland et al., 2006). An additional 12 bp duplex of B-DNA was added to the end of each *res* site in order to better visualize the path of the DNA as it exits the complex (Figure 6).

This tightly constrained docking procedure generates a model of the synaptic complex that satisfies several critical requirements. The two *res* duplexes intertwine to form three plectonemic (−) supercoils, in agreement with the topology of recombination (Figure 1B) (Rowland et al., 2002); a minor steric clash between the right end of the site II DNA and the segment it crosses (the right end of site I) could be relieved by small adjustments to the flexible protein-DNA complexes. The configuration of the site II duplexes, wrapped around each other near the center of the regulatory complex, aids the extreme condensation of DNA supercoiling (three plectonemic supercoils in ∼172 bp). There are no steric clashes between the protein components of the synaptic complex model, yet nearly the entire length of the DNA in the complex is contacted by at least one of the bound proteins, in agreement with previous footprinting data (Rowland et al., 2002).

## Discussion

This work describes the structural characterization of a serine recombinase regulatory complex, revealing the basis for its catalytic inactivity. An interface between the DNA-binding domains (CTDs) is shown to mediate synapsis of the regulatory sites. The resulting synaptic tetramer (Figure 3B) is the keystone of a model for the recombination-proficient synaptic complex (Figure 6), assembled entirely from available crystal structures and supported by mutational data.

### Architecture of the Synaptic Complex

Sin is a remarkably versatile protein that can bind as a dimer to direct or inverted DNA repeats (Figure 1A) as well as form several different protomer-protomer interfaces. Three interfaces revealed by the Sin-site II structure are involved in assembly and function of the regulatory complex: (1) the site II dimer interface, mediated by the E helices (Figure 2A), which resembles the γδ resolvase-site I presynapsis dimer interface (Figure 2B) (Yang and Steitz, 1995); (2) the site II synapsis interface, mediated by the CTDs (Figures 3B and 4); and (3) an interface involving F52 and R54 (Figure 3C and Figure S1), implicated in regulatory contacts between site I- and site II-bound tetramers. The fourth interface seen in our crystals, shown in Figure 3A, may reflect a transient interaction that occurs between site I-bound Sin dimers before the dimers undergo major conformational changes required to assemble an active site I tetramer similar to that of γδ resolvase (Kamtekar et al., 2006; Li et al., 2005).

The unexpected CTD-mediated synapsis interface at site II holds the DNA duplexes in close proximity (<10 Å) and is the basis for the architecture of the proposed synaptic complex (Figure 6). In contrast, to the best of our knowledge, all previously published models for the Tn3/γδ resolvase and Sin synaptic complexes have placed the N-terminal domains at the core of the regulatory synapse and either fail to readily accommodate all data or rely on hypothetical protein-protein interfaces (Murley and Grindley, 1998; Rice and Steitz, 1994a; Rowland et al., 2002; Sarkis et al., 2001; Stark et al., 1989). Although the NTD-mediated crystal packing of Sin tetramers, through interactions of residues F52 and R54, is similar to the arrangement of synaptic tetramers postulated by Sarkis et al. for γδ resolvase, an NTD-mediated synaptic tetramer at Sin site II (Figure 3A) would define a right-handed (+) node instead of the left-handed (−) node predicted by topological experiments and would provide no role for the CTD residues identified in the site II synapsis screen. As described below, we propose that F52 and R54 do mediate tetramer-tetramer interactions, but within a different overall structural context.

The Sin synaptic complex harnesses DNA bending by Sin and IHF to trap a high density of plectonemic supercoiling in the relatively short (86 bp) *res* sites. Each site II duplex is bent away from the NTDs of the Sin dimer by insertion of an E helix into the minor groove, a mechanism also used by γδ resolvase to bend the site I duplex away from its NTDs. When two Sin dimers synapse via the CTD interface, this allows the duplexes to wrap tightly around one another (Figure 3B). Previous synapse models that utilize only NTD-mediated interfaces require the DNA bound at the accessory sites (site II in the Sin system or sites II and III in the Tn3/γδ resolvase systems) to bend toward the NTDs, a conformation opposite to that seen in the Sin and γδ resolvase crystal structures. In our model for Sin, the sharp IHF-induced bend in the site I-site II spacer brings the site Is into proximity, such that they could be synapsed by a catalytic tetramer resembling that of γδ resolvase. If the Sin-site II interface were NTD mediated, binding of IHF would cause the site I-site II spacers to bend away from each other, making it difficult to construct a model that synapses site Is and traps the predicted supercoiling nodes (Figure S3).

Many resolution systems use “Sin-like” *res* sites (Rowland et al., 2002), e.g., the well-studied β recombinase (Canosa et al., 1996, 2003), and are likely to utilize a CTD synapsis interface at site II. We are currently investigating whether the Sin regulatory site tetramer can serve directly as a model for Tn3/γδ resolvase regulatory site interactions. One reasonable hypothesis is that site III of Tn3/γδ *res* (Figure 1A) plays the role of site II in the Sin synaptic complex (Rowland et al., 2002) and that site II of Tn3/γδ *res* is primarily a site of DNA bending (Salvo and Grindley, 1988; Soultanas and Halford, 1995). There is a small hydrophobic patch on the γδ resolvase CTD (centered on L159, M154, and M168), and one can use it to model a CTD-mediated synaptic tetramer at site III. Although site III has inverted rather than direct repeats (Figure 1A), the spacing at site III could permit synergistic interactions between pairs of CTDs to give a tetramer with approximate 222 symmetry. However, in the absence of supporting data, such modeling is highly speculative.

Our data reveal that the small “DNA-binding” domain of a resolvase has a previously overlooked function of mediating synapsis between DNA sites. In addition to being found in other serine recombinases, similar small 3-helix DNA-binding domains are also present in many DDE transposases, and recent structures of mariner family transposases (Richardson et al., 2006; Richardson et al., 2007; Watkins et al., 2004) provide further evidence that these domains may often have an additional function in DNA synapsis.

### Structural Basis for Regulation of Recombination

The model for the synaptic complex that is supported by the structural and genetic data presented here is fundamentally different from previous proposals and provides a new perspective on how communication between catalytic and regulatory modules could be achieved through direct interactions.

We have shown evidence that residue R54 of Sin is involved in communication between catalytic and regulatory subunits (Figure 5C); genetic data implicate both F52 and R54 (S.J.R., unpublished data). In γδ resolvase, the structurally analogous 2-3′ interface residues E56 and R2 (Figure 3C and Figure S1) have a key regulatory role (Hughes et al., 1990; Murley and Grindley, 1998). In the model as shown in Figure 6B, the N-terminal domains of the site I- and site II-bound recombinase subunits are in close proximity. A small rotation of the site II-bound N-terminal domains about a hinge point within helix E would bring residue 42 and the preceding flexible turn into contact with residues R2 and E56 of the γδ resolvase protomer modeled at site I of the same *res* site. However, there is currently no experimental evidence for an interface involving Sin residues 37–42. We favor an alternative scenario in which the symmetric crystallographic interface involving residues F52 and R54 (Figure 3C and Figure S1) is modeled between sites I and II of each *res* site (Figure 6C). This requires the following adjustments to the model of Figures 6A and 6B: (1) the NTDs of the site I-bound γδ resolvase tetramer are replaced with Sin NTDs; (2) the NTDs of the site II-bound dimers (up to the flexible hinge in the E helix) are arranged to make symmetric F52/R54 contacts with the site I NTDs; and (3) the remainder of the site II tetramer complex (comprising the C-terminal portions of the E helices, the DNA, and the CTDs) is translated ∼15 Å away from the site I tetramer (as a single rigid body). Unlike the model of Figures 6A and 6B, in which the intact crystal structures were treated as rigid bodies and DNA continuity was maintained, this “direct interaction” model (Figure 6C) requires the above adjustments to the structures and results in small gaps (5–10 Å) in both the protein (at the hinge regions of the site II-bound protomers) and the DNA (such that the spacer bound by IHF does not completely bridge the site I-site II gap). However, given the known structural plasticity of Sin, and the variety of DNA bend angles that can be induced by IHF and HU (Swinger et al., 2003; Swinger and Rice, 2004), formation of such a structure at a point along the recombination pathway seems entirely plausible.

Direct interactions between the regulatory and catalytic modules are thought to activate the protomers bound to site I by promoting the transition from two inactive dimers to the activated, synapsed tetramer. The direct interactions in our model could promote tetramerization by “extreme mass action”—that is, by bringing the two site I-bound dimers into close proximity in the optimal orientation for tetramer formation. They could also allosterically promote the required conformational changes within the individual monomers contacted, although the exact mechanism of such allostery is unclear at this time. Hin, a DNA invertase that catalyzes gene switching in *Salmonella*, is proposed to require direct contacts with Fis bound at the enhancer site to activate DNA cleavage at the crossover site (Merickel et al., 1998). However, not all systems that assemble an interwound synapse rely on direct contacts between the catalytic and regulatory machinery: *E. coli* XerCD, when it acts on plasmids, requires a synaptic complex that traps three negative supercoils, but the accessory proteins that determine reaction topology do not interact directly with XerCD (Bregu et al., 2002; Colloms et al., 1997).

Regulation of Sin catalysis by the accessory sites imparts exquisite directionality to the system and may prevent the inappropriate formation of DNA double-strand breaks or recombination between *res* sites that are improperly oriented or on different plasmids. In contrast, the site-specific recombinases that have traditionally been used to manipulate eukaryotic genomes, Cre and Flp, catalyze recombination in both the forward and reverse directions and without regard to substrate topology. Efforts to design more efficacious tools for genome engineering (Calos, 2006) may be aided by our insights into the structures used by Sin, β recombinase (Gronlund et al., 2007), and other serine recombinases to direct and regulate catalysis.

## Experimental Procedures

### Sample Preparation

Wild-type His_6_-tagged pI9789 Sin was expressed from pSA1122 (Rowland et al., 2005) in Rosetta (DE3)[pLysS] cells (Novagen). The cells were grown at 37°C in Luria-Bertani medium containing 50 μg/ml kanamycin. When the OD_600_ reached ∼0.6, the cells were induced with 0.5 mM isopropyl-β-D-thiogalactopyranoside (IPTG), grown for an additional 3 hr, and then harvested by centrifugation. The cell pellet was resuspended in buffer A (25 mM TRIS [pH 7.5], 100 mM NaCl, 2.5 mM EDTA, 10 mM β-mercaptoethanol, and protease inhibitor cocktail [Boehringer Complete]). Lysozyme was added to 200 μg/ml, and the mixture was incubated on ice for 20 min, sonicated, then centrifuged at 38,000 × g for 30 min. Sin remained in the pellet, which was subsequently resuspended and centrifuged in buffer B (buffer A except 400 mM NaCl) and then buffer C (buffer A plus 1% Triton X-100). The pellet was then resuspended in buffer D (25 mM TRIS [pH 7.5], 1 mM EDTA, 1 mM β-mercaptoethanol, and 6 M urea) and centrifuged. The denatured Sin is soluble and was loaded on an SP column (Amersham Biosciences) and eluted with buffer E (buffer D except 1 M NaCl). Pooled fractions were dialyzed into buffer F (20 mM Na_2_HPO_4_/NaH_2_PO_4_ [pH 7.5], 6 M urea, and 0.5 M NaCl) and then loaded on a Hi-Trap chelating column (Amersham) charged with NiCl_2_ and eluted with buffer G (buffer F plus 0.5 M imidazole). Pooled fractions were dialyzed into buffer D, loaded on a Mono S column (Amersham), and eluted with buffer E. Fractions containing full-length Sin were dialyzed into 25 mM TRIS (pH 7.5), 0.5 mM EDTA, 400 mM (NH_4_)_2_SO_4_, and 20% glycerol and concentrated to ∼20 mg/ml in centrifugal filters (MWCO 10000, Amicon). DNA oligonucleotides were from Integrated DNA Technologies (Coralville, IA) or the Keck Oligonucleotide Synthesis Facility (Yale University) and were annealed in 100 mM NaCl.

### Crystallization and Structure Determination

Crystals were grown with the hanging drop vapor diffusion method. WT Sin and DNA were mixed in a 2:1.25 molar ratio (final concentration ∼4 mg/ml) in a buffer containing 25 mM TRIS (pH 7.5) and 75 mM (NH_4_)_2_SO_4_ and incubated for 20 min at room temperature before being mixed in a 1:1 ratio with well solutions containing 25 mM TRIS (pH 7.5) and 100–125 mM (NH_4_)_2_SO_4_. Hexagonal crystals appeared in 2–3 days and grew to full size (125 × 125 × 400 μm) in 5–7 days. Crystals were cryoprotected in well solutions containing 20% glycerol and frozen in liquid N_2_. Diffraction data were collected at the SBC beamline 19-ID at the Advanced Photon Source (Argonne, IL) and processed with the HKL2000 suite (Otwinowski and Minor, 1997). Data were collected from crystals containing the native DNA as well as from complexes in which T's in the sequence were replaced with 5-iodo-dU or 5-bromo-dU (Figure 2). Phases were determined from SIRAS data from iodine-containing crystals and MAD data from the bromine crystals. All crystals were isomorphous and belong to space group P6_5_, with two DNA-bound Sin dimers per asymmetric unit (a.s.u.) and a solvent content of 79% (v/v).

The positions of eight of the ten iodine atoms in the a.s.u. were determined with SHELX-97 (Sheldrick, 1998) and then used in SOLVE (Terwilliger and Berendzen, 1999) to locate 22 of the 26 bromine atoms. Careful analysis of the bromine and iodine anomalous difference maps showed no peaks consistent with additional duplexes in the a.s.u. Phases were improved through cycles of solvent flattening and phase extension, with and without averaging about a two-fold noncrystallographic (ncs) axis that relates the two DNA-bound dimers. The data are anisotropic, and the reported resolution limit of 3.21 Å is a conservative compromise based on the 3.34–3.21 Å shell for which, over the entire native data set, 〈I〉/〈σ_I_〉 is ∼2.9. The diffraction begins to fall off rather steeply after 3.5 Å along a^∗^ and b^∗^ but extends past 3.1 Å along c^∗^ (anisotropic scale factors are B11 = −13.5, B22 = −13.5, and B33 = 27).

An initial model was constructed with selected secondary structural elements from the γδ resolvase-site I dimer structure (1GDT), and the register of the DNA was assigned by using the heavy atom positions. The structure was refined using cns_solve (Brunger et al., 1998), with a combination of two-fold and four-fold ncs restraints (several surface residues involved in protein-protein contacts were excluded from the restraints, as were helix D′ and the turn connecting it to helix E). The program O (Jones et al., 1991) was used for model building and PyMOL (DeLano, 2002) was used for figures. The final model contains 768 of 836 Sin residues and 62 bp of DNA and was refined to an R_working_ of 26.7 and an R_free_ of 29.1 with good stereochemistry (Table 1 and Figure S4). In all four monomers, one loop (35–41) and nine residues at the C terminus (containing the His_6_ tag) are absent from the model, as are residues 130–131 from two of the monomers.

### Mutagenesis of Sin and Selection of Site II Synapsis Mutants

The entire coding sequence for WT pI9789 Sin in pSA9944 was randomly mutagenized by PCR in the presence of either 20 μM 8-oxo-G or 20 μM dPTP (Zaccolo et al., 1996), and two mutant libraries (7.1 and 7.4; both ∼6 × 10^5^) were generated. pSA9944 is similar to pSA9903 (Rowland et al., 2005) except that the Sin ORF contains additional restriction sites and encodes a C-terminal His_6_ tag. Sublibraries of mutants that are defective in site II synapsis (7.1S, ∼1900; 7.4S, ∼2500) were selected by using a complementation assay. Libraries 7.1 and 7.4 were transformed into *E. coli* DS941 (*recF*, *galK*) containing the test substrate pSB386 (pSC101 based; Figure 5A), and these new strains were transformed with pMS173 (pACYC184 based), which expresses a mutant of Cre (M44I/R121Q/E123G/S205C/D232E/I309L/T316P; to avoid the toxic effect of WT Cre on cell growth in this assay). Synapsis-defective mutants of Sin are unable to block Cre-*loxP* recombination; therefore, the reporter gene (*galK*) is lost, and colonies are white (rather than red) on MacConkey/galactose plates that select for all three plasmids. Sublibraries 7.1S and 7.4S were then screened for mutants that can bind at site II, in a complementation assay using pSB398 (pSC101-based), in which site II (underlined) overlaps a promoter (lower case) for a *galK* gene: ttgacaGCTTATCAGAGCTCAAACGtatgatTAGGGTGTATATTAATT. Binding by Sin represses the promoter and reduces *galK* expression, resulting in pale pink (rather than red) colonies on selective MacConkey/galactose indicator plates. Of the 13 single mutations characterized (Figure 5B), seven were isolated from both libraries.

### In Vivo Recombination Assays

The recombination substrates pSB(*resH* × *resH*) (here called “*res* × *res*”) and pSB(site I × site I), and the complementation assay, have been described (Rowland et al., 2002). In brief, resolution deletes a reporter gene (*galK*), resulting in white colonies on selective MacConkey/galactose indicator plates; red colonies indicate no resolution or a low rate of resolution. Pooled colonies (>100) from the plate assay were grown in liquid culture, and plasmid DNA was analyzed by agarose gel electrophoresis to confirm the nature of the recombination products (data not shown). To estimate percentage of resolution, plasmid DNA was transformed into *E. coli* DS941, selecting for the substrate replicon; unresolved and resolved forms give red and white colonies, respectively, on indicator plates (>2000 counted for each assay). Where necessary (for the *res* × *res* reactions of I100T/K161R, I100T/E170G, I100T/E170K, and I100T/N186S), a quantitation artifact due to cotransformation of the Sin-expressing plasmid was prevented by transforming the DNA into *E. coli* DS941 containing pAT6Δ (similar to pSA9944, but expressing WT Tn3 resolvase).

### Selecting a Suppressor of S153T

In the plasmid expressing I100T/S153T, the sequence encoding residues Q160–H209 (inclusive) was replaced by corresponding randomly mutagenized sequence from library 7.1 or library 7.4, to give two new libraries. Mutants that could recombine pSB(*resH* × *resH*) (see above) were then selected; nine isolates, derived from both libraries, were I100T/S153T/H166R.

## Figures and Tables

**Table 1:** Crystallographic Statistics

Data Collection Statistics	Native	Br (Peak)	Br (Edge)	Iodine				
Wavelength (Å)	0.91977	0.91977	0.91988	1.77				
Resolution range (Å)	50−3.21	50−3.62	50−3.94	50−3.4				
Unique reflections	49,946	34,786	26,783	43,361				
R_merge_^a^	0.13	0.15	0.12	0.15				
〈I〉/〈σ_I_〉^b^	9.1 (2.9)	10.8 (2.5)	8.8 (2.2)	13.5 (1.7)				
Redundancy	7.9 (7.5)	17.9 (16.5)	4.6 (4.5)	12.9 (7.0)				
Completeness (%)	97.5 (95.6)	99.9 (99.9)	99.9 (99.9)	99.9 (99.9)				

Phasing Statistics								

Phasing power	Resolution shells (Å)							
(Centric/acentric)	50−10.7	10.7−7.09	7.08−5.61	5.60−4.78	4.77−4.24	4.23−3.84	3.83−3.54	Overall
Bromine	0.6/1.21	0.86/1.22	0.93/0.94	0.50/0.76	0.38/0.60	−/0.47	−/0.43	0.65/0.92
Iodine	0.47/0.82	0.46/0.66	0.49/0.53	0.24/0.40	0.24/0.30	0.27/0.22	0.16/0.25	0.39/0.43
Figure of merit	0.67	0.68	0.58	0.47	0.40	0.24	0.10	0.41

Refinement Statistics								

Space group	P6_5_							
Unit cell dimensions (Å)	a = b = 132.5, c = 315.1							
Solvent content (%)	79							
Resolution range (Å)^c^	50−3.21							
Number of atoms								
Protein	6164							
DNA	2530							
Ion (sulfate)	2							
Rmsds								
Bond length (Å)	0.01							
Bond angle (°)	1.35							
R_working_/R_free_^d^	26.7/29.1							
Ramachandran analysis^e^								
Most favored	83%							
Additionally allowed	16%							

Values in parentheses are data from highest resolution shell. Figure of merit values were calculated prior to density modifications.

a R_merge_ is Σ_j_|I_j_ − 〈I〉|, where I_j_ is the intensity of an individual reflection and 〈I〉 is the mean intensity for multiply recorded reflections.

b 〈I〉/〈σ_I_〉 is the mean intensity divided by the mean error.

c The native data set is anisotropic; the reported resolution limit represents an average of the approximate resolution limits within cones along each axis.

d R_working_ is Σ||F_o_| − |F_c_||/Σ|F_o_|, where F_o_ is an observed amplitude and F_c_ is a calculated amplitude; R_free_ is the same statistic calculated over a subset of the data that have not been used for refinement.

e Ramachandran analysis from PROCHECK (Laskowski et al., 1993).

## Accession Numbers

The atomic coordinates of the Sin-site II complex have been deposited in the Protein Data Bank under accession number 2R0Q.

## Supplemental Data

Document S1. Five Figures

Movie S1. The Synaptic Complex Model Shown in Figures 6A and 6B Is Rotated 360° about a Vertical Axis The model was constructed by rigid-body docking of the Sin-site II tetramer from Figure 3B with a γδ resolvase site I tetramer (Li et al., 2005) and IHF-DNA complexes (Rice et al., 1996). Extra base pairs from each complex were overlapped to attain the exact length and register of the 86 bp *res* site. The IHF site is located precisely midway between sites I and II, as required for efficient recombination (Rowland et al., 2006). The *res* duplexes in the resulting model intertwine to form three (−) supercoiling nodes, in agreement with the topology of recombination (Rowland et al., 2002). No steric clashes between protein components are observed, yet nearly the entire length of the res duplexes is contacted by protein, as predicted by footprinting experiments (Rowland et al., 2002). (References can be found within the main Supplemental Data file.)
