# Supplementary material for: Architecture of a Serine Recombinase-DNA Regulatory Complex
Source: Mol Cell. 2008 Apr 25;30(2):145–55. doi: 10.1016/j.molcel.2008.02.023 (PMC2428073; doi:10.1016/j.molcel.2008.02.023)

Molecular Cell, Volume *30*

Supplemental Data

Architecture of a Serine Recombinase-DNA Regulatory Complex

Kent W. Mouw, Sally-J. Rowland, Mark M. Gajjar, Martin R. Boocock, W. Marshall Stark, and Phoebe A. Rice

Supplemental Figure Legends

Figure S1: Comparison of a pseudo-symmetric crystal packing interface observed in the Sin structure with the analogous 2-3' interface of γδ resolvase (Rice and Steitz, 1994b) Only the N-terminal domains of the monomers contributing residues to the interface are shown. Residues F52 and R54 are shown for Sin, and residues R2, R32, K54, and E56 are shown for γδ resolvase. Mutation of these residues affects communication between catalytic and regulatory sites within the synaptic complex (see text) (Hughes *et al.*, 1990; Murley and Grindley, 1998; Rice and Steitz, 1994a). R54 (orange) and F52 of Sin have roles in the interface analogous to R2 (orange) and E56 of γδ resolvase.

Figure S2: Sequence alignment of Sin and related serine recombinases

The top three rows show secondary structural assignments of serine recombinases for which crystal structures are available: γδ resolvase (Yang and Steitz, 1995), Hin invertase (Feng *et al.*, 1994), and Sin. All structural and genetic work in this study involved Sin-pI9789. Resolvases known or inferred to act on "Sin-like" *res* sites include β recombinase, Tn1546 and pXO1 resolvases, and all seven variants of Sin. Resolvases

known or inferred to act on *res* sites with three dimer binding sites include those from γδ, Tn3, Tn21 and TnARS1 and the ParA resolvase from RP4. Additional domains of the large serine recombinases (CcrA & ϕC31) and IS607 transposase have been excluded from the alignment and are represented by "hth" for helix-turn-helix domain or "ctd" for C-terminal domain. Residues are color-coded by chemical property. Residues that were highlighted in the screen for site II synapsis mutants are labeled with an asterisk (*). The position of a supressor mutation, H166R, that restores site II synapsis in a synapsis-deficient S153T background, is labeled with a filled circle (●). Positions of activating mutations are labeled with diamonds (◇) and positions of residues that are implicated in site I – site II communication are labeled with open circles (**O**) (see text, Fig 5). The position of the sharp kink in Sin helix E is denoted with a wedge (▾).

<u>Figure S3:</u> Attempts to model a hypothetical NTD-mediated site II synapsis interface (stereo view)

A. The NTD-mediated Sin site II synaptic tetramer (Fig 3A) was docked with IHF-DNA complexes (extra basepairs from each complex were overlapped to attain the proper DNA register) in order to visualize the resulting architecture of the hypothetical regulatory module. The ends of the DNA bound by IHF are separated by approximately 180 Å, much further than the ~90 Å that separates ends of the site I duplexes bound by the γδ resolvase catalytic tetramer, which Sin is thought to resemble. The DNA duplexes form a single (+) right-handed crossing node, whereas the synaptic complex is predicted to trap 3 (-) left-handed nodes.

B. One of the Sin dimers has been rotated 180º about the NTD interface. Although the structure now defines a (-) node crossing at site II, the DNA ends are separated by 120 Å, substantially further than the ~90 Å predicted to separate ends of the site Is in the catalytic tetramer, and it is not clear how two additional (-) nodes could be trapped.

Figure S4: Ramachandran plot of final Sin model generated using PROCHECK (Laskowski *et al.*, 1993).

Figure S5: Experimental electron density maps (no model phase information)

A. Bromine anomalous difference map (contoured at 7.0σ) showing positions of the 5-Br-dU bases used in phasing. The positions of the peaks were used to unambiguously assign the register of the DNA.

B. Stereo-view experimental electron density map of the site II-bound Sin dimer. The map is contoured at 1.0σ and is color-coded to show all density within 3.5 Å of all atoms of each of the Sin monomers (blue, green) and the site II duplex (orange). The interdomain linker of the green monomer is clearly visible.

C. The experimental electron density map (contoured at 1.0σ) shows clear density for individual β-strands within the Sin NTD.

Supplemental References

Feng, J. A., Johnson, R. C., and Dickerson, R. E. (1994). Hin recombinase bound to DNA: the origin of specificity in major and minor groove interactions. Science *263*, 348-355.

Hughes, R. E., Hatfull, G. F., Rice, P., Steitz, T. A., and Grindey, N. D. F. (1990). Cooperativity mutants of the γδ resolvase identify an essential interdimer interaction. Cell *63*, 1331-1338.

Laskowski, R. A., MacArthur, M. W., Moss, D. S., and Thornton, J. M. (1993). PROCHECK: a program to check the stereochemical quality of protein structures. J. Appl. Crystallog. *26*, 283-291.

Li, W., Kamtekar, S., Xiong, Y., Sarkis, G. J., Grindley, N. D. F., and Steitz, T. A. (2005). Structure of a synaptic γδ resolvase tetramer covalently linked to two cleaved DNAs. Science *309*, 1210-1215.

Murley, L. L., and Grindley, N. D. F. (1998). Architecture of the γδ resolvase synaptosome: oriented heterodimers identify interactions essential for synapsis and recombination. Cell *95*, 553-562.

Rice, P. A., and Steitz, T. A. (1994a). Model for a DNA-mediated synaptic complex suggested by crystal packing of γδ resolvase subunits. EMBO J. *13*, 1514-1524.

Rice, P. A., and Steitz, T. A. (1994b). Refinement of γδ resolvase reveals a strikingly flexible molecule. Structure *2*, 371-384.

Rice, P. A., Yang, S., Mizuuchi, K., and Nash, H. A. (1996). Crystal structure of an IHF-DNA complex: a protein-induced DNA U-turn. Cell *87*, 1295-1306.

Rowland, S.-J., Boocock, M. R., and Stark, W. M. (2006). DNA bending in the Sin recombination synapse: functional replacement of HU by IHF. Mol. Microbiol. *59*, 1730-1743.

Rowland, S.-J., Stark, W. M., and Boocock, M. R. (2002). Sin recombinase from *Staphylococcus aureus*: synaptic complex architecture and transposon targeting. Mol. Microbiol. *44*, 607-619.

Yang, W., and Steitz, T. A. (1995). Crystal structure of the site-specific recombinase γδ resolvase complexed with a 34 bp cleavage site. Cell *82*, 193-207.

Figure S1

Sin γδ resolvase

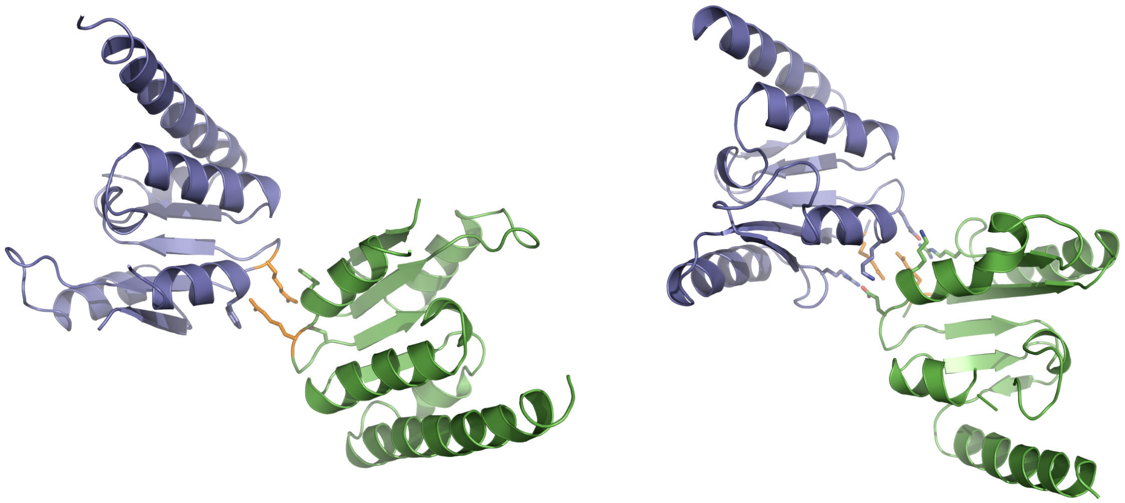

Figure S2

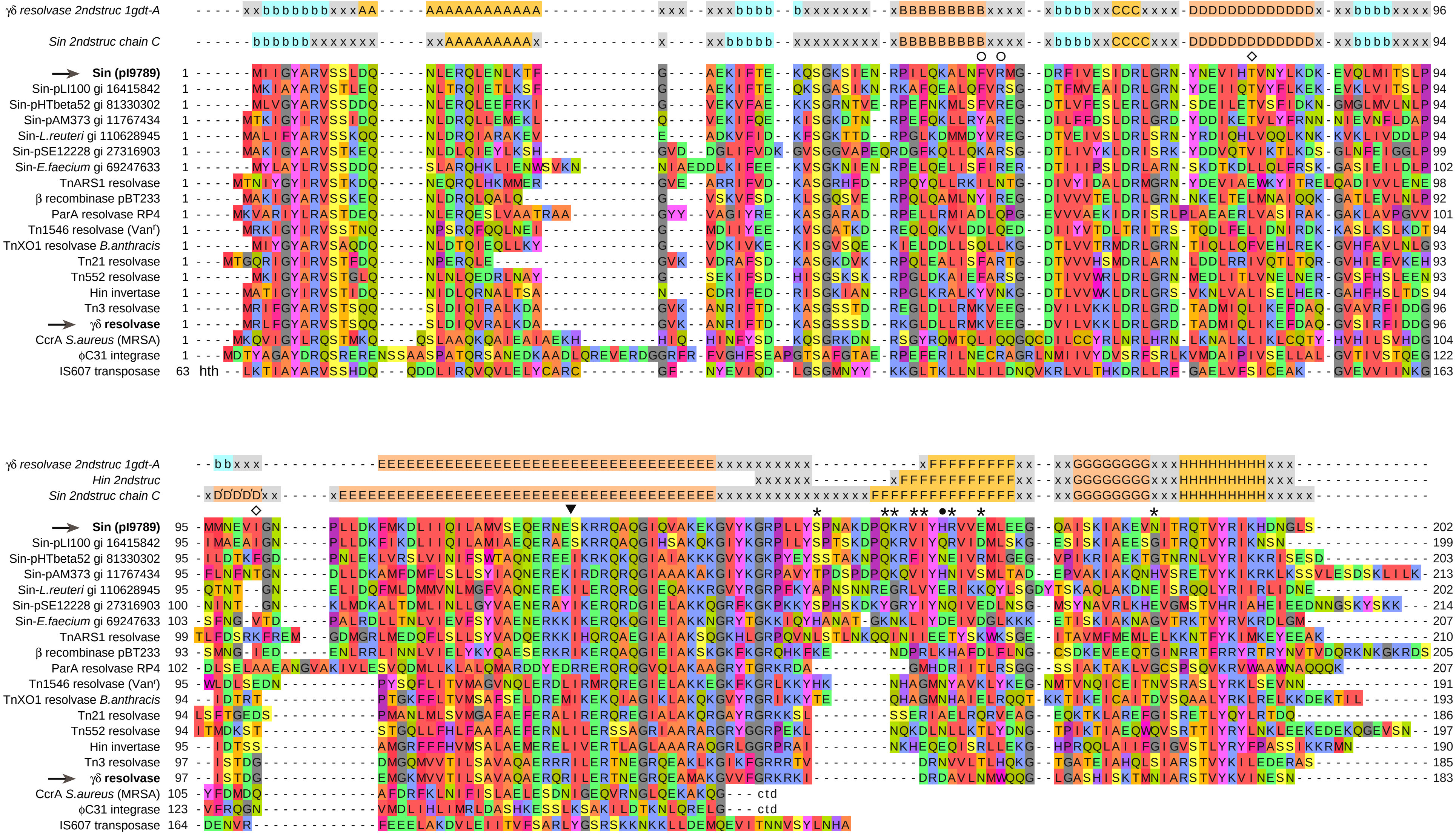
γδ resolvase 2ndstruc 1gdt-A
Sin 2ndstruc chain C
Sin (pI9789)
Sin-pLI100 gi 16415842
Sin-pHTbeta52 gi 81330302
Sin-pAM373 gi 11767434
Sin-L.reuteri gi 110628945
Sin-pSE12228 gi 27316903
Sin-E.faecium gi 69247633
TnARS1 resolvase
β recombinase pBT233
ParA resolvase RP4
Tn1546 resolvase (Van^r)
TnXO1 resolvase B.anthracis
Tn21 resolvase
Tn552 resolvase
Hin invertase
Tn3 resolvase
γδ resolvase
CcrA S.aureus (MRSA)
ϕC31 integrase
IS607 transposase
hth
Hin 2ndstruc
ctd

Figure S3

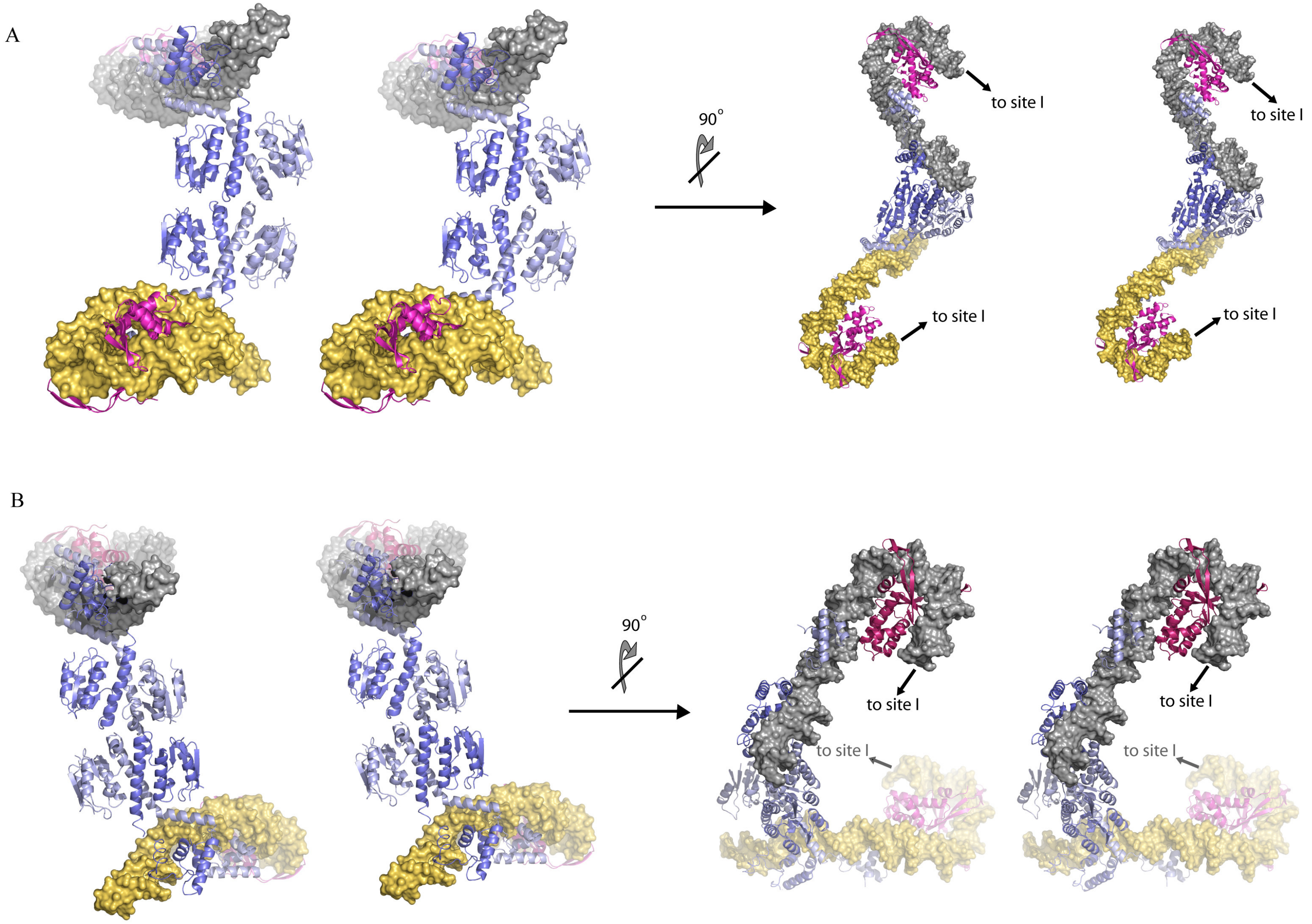
A
90°
to site I
to site I
to site I
to site I
B
90°
to site I
to site I
to site I
to site I

Figure S4

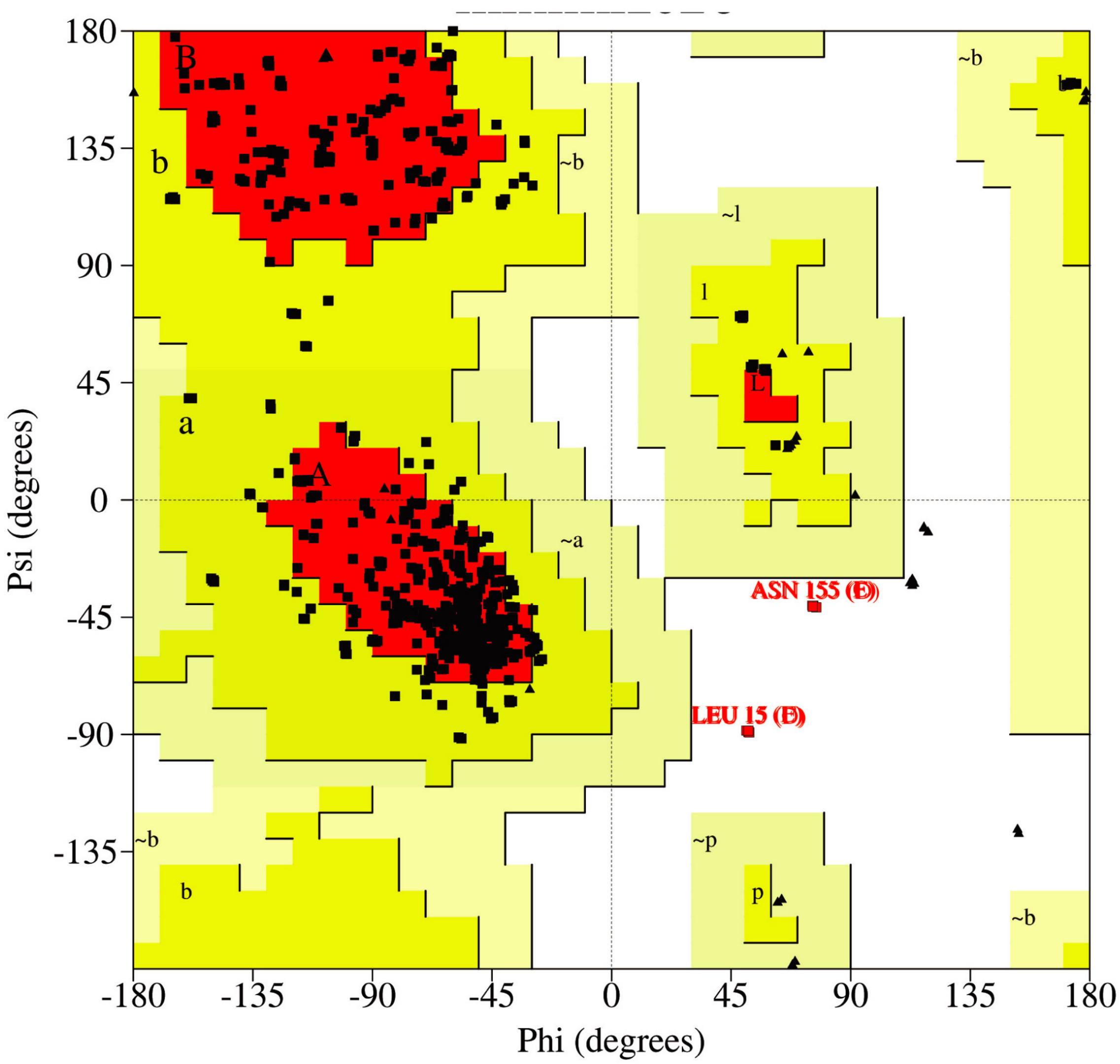

Plot statistics

Residues in most favoured regions [A,B,L]	566	82.3%
Residues in additional allowed regions [a,b,l,p]	114	16.6%
Residues in generously allowed regions [~a,~b,~l,~p]	0	0.0%
Residues in disallowed regions	8	1.2%
	----	------
Number of non-glycine and non-proline residues	688	100.0%
Number of end-residues (excl. Gly and Pro)	142	
Number of glycine residues (shown as triangles)	40	
Number of proline residues	24	

Total number of residues	894	

Figure S5

A

B

C

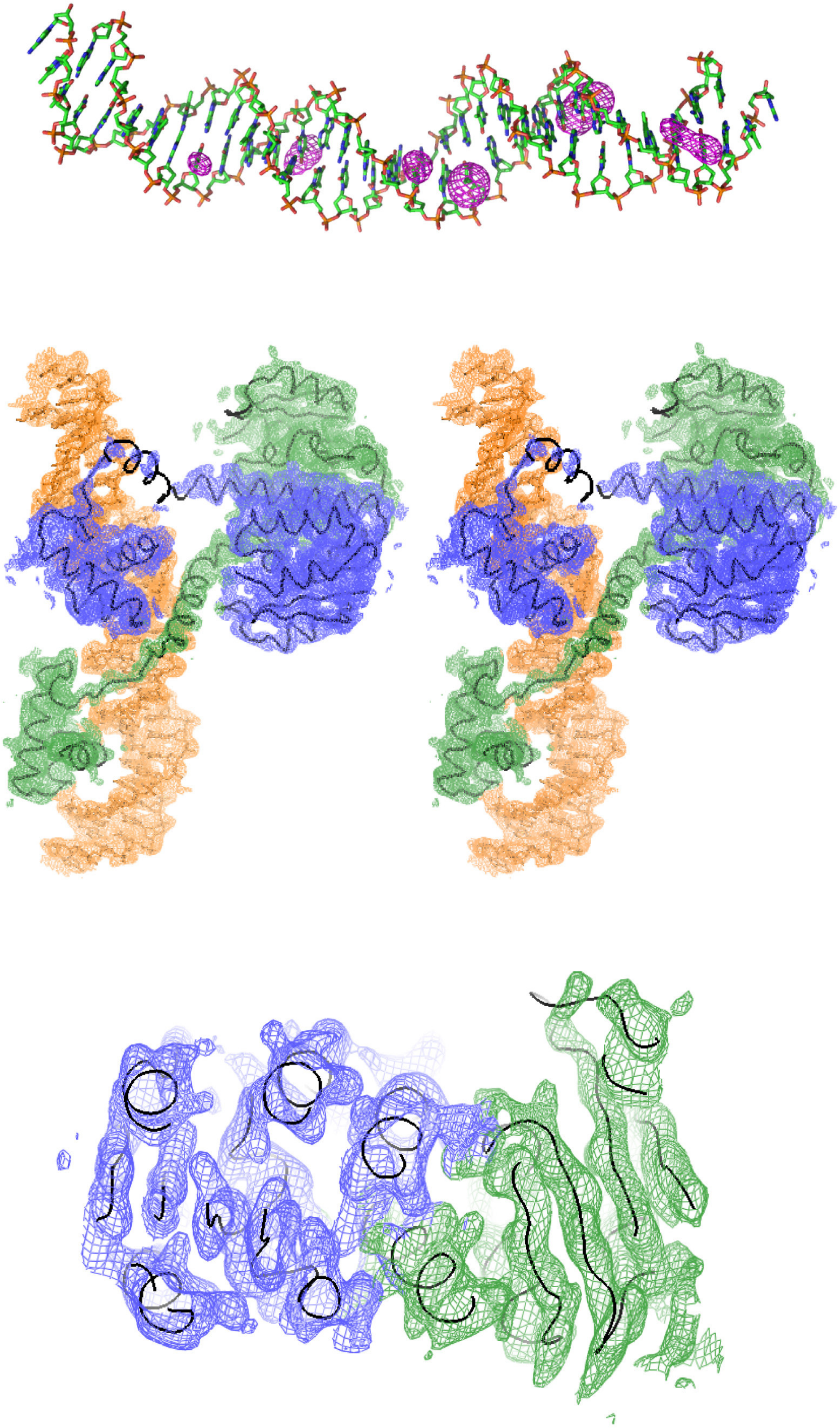

Supplement: Document S1. Five Figures [file mmc1.pdf]
